# Supplementary material for: Development of a proposed solutions box for improving nutritional behaviors of female school students: Protocol of a mixed-method study
Source: PLoS One. 2025 Sep 24;20(9):e0329671. doi: 10.1371/journal.pone.0329671 (PMC12459775; doi:10.1371/journal.pone.0329671)
Supplement: Attachment — Submitted filename: Review - PlosOne.docx [file pone.0329671.s001.docx]

The authors have presented a mixed method—qualitative and quantitative—to detect the underlying causes of unhealthy nutritional behaviors in adolescent female students in Khouzestan, Iran. The structured presented follows the rule of logic and shows promising aspects with an important health issue in Iran. However, the following concerns might need to be addressed.

**A) Writing/Structure**

1) Line 83: ‘inappropriate’ is not a technical term when talking about eating behaviors.

2) It seems that some words have been missed in Figure 1 under ‘NUTRIBOX.’

3) Revise the manuscript with regard to its writing. I specifically noticed some misuses of capitalization.

**B) Methods/Fundamentals**

1) Line 191: Are ‘tape recorders’ still available for use? Are they viable options regarding the existence of digital recorders for safe storage of data?

2) Sampling method need more explanation than just to achieve ‘maximum diversity.’ The authors need to specify what schools will be chosen and why. For instance, are schools chosen geographically, based on socio-economic features shared by residing students, etc.? And the method needs to be specified; convenience sampling, cluster sampling, etc.

3) Line 266-267: The fact that the researchers ‘will try to select more diverse participants’ is not the assurance that the authors presume. A detailed plan must be laid out to ensure including students from various backgrounds. I specially recommend that socioeconomic status (SES) of attendees be taken as the main criteria for inclusion of students. In other words, should participants from a specific background be selected—a real possibility with the present scheme—the outputs will lose generalizability. Authors must be aware that eating/nutritional behaviors are widely affected by environmental factors and the present scheme seems to skew towards a major selection bias.

4) Knowingly, gender and gender identification, as well as issues dealing with sexual activities, commence in adolescence. These subjects, with the potential mediatory impact of distortions in body image or competitive behaviors to create a ‘fitter’ body shape, could modify the individual’s perception of healthy body shape, and thus, their eating behaviors. Are the authors—interviewers—open to discuss these matters with the interviewees? If so, the manuscript needs to include them.
