# Supplementary material for: Development of a proposed solutions box for improving nutritional behaviors of female school students: Protocol of a mixed-method study
Source: PLoS One. 2025 Sep 24;20(9):e0329671. doi: 10.1371/journal.pone.0329671 (PMC12459775; doi:10.1371/journal.pone.0329671)
Supplement: Attachment — Submitted filename: Response to reviwer.doc [file pone.0329671.s004.doc]

**Rebottle letter**

**21.02.2025**

**Dear Editor-in-Chief of PLOS ONE**

Many thanks for the precise comments on our MS: “**Development of a Proposed Solutions Box for Improving Nutritional Behaviors of Female School Students: Protocol of a Mixed-Method Study.** We greatly appreciate the time and effort you have dedicated to providing us with such valuable feedback.

- We have carefully considered all of the reviewers' comments and have addressed them in tabulated format, which have submitted alongside the revised MS.
- Accordingly, all necessary changes to the MS were **highlighted** in **green**.

Once again, we thank you for your thoughtful review and for helping us to improve our MS.

**Sincerely,**

**Fatemeh Zarei**

Corresponding author

[f.zarei@modares.ac.ir](mailto:f.zarei@modares.ac.ir)

**Response to Reviewers**

**Dear Reviewers,**

We sincerely appreciate your valuable time and insightful comments on our manuscript. Your feedback has significantly improved the quality of our work. We have carefully addressed all your suggestions and revisions, ensuring that each point has been thoroughly considered.

Thank you for your constructive review and support.

**Best regards**

Corresponding Athours

| **#Reviewer 1** | **Response** | |
| --- | --- | --- |
| **Development of a Proposed Solutions Box for Improving Nutritional Behaviors of Female 2 School Students: Protocol of a Mixed-Method Study** Research like this is worth pursuing and provides deep insight into preventive medicine but some major recommendations can be found below Overall editing of the text is recommended to make the content structurally understandable and simplified for better readability. Such as shortening the sentences and improving transitions between ideas helps readers to get a better your point and understand the logic and rationale of your study. | Thank you for your thoughtful feedback. We have carefully addressed your comments and made the necessary revisions, as detailed below. | |
| **Comments & Suggestions** | **Response** | **Action** |
| **Abstract:** The Aim part fails to properly state and justify the purpose and necessity of conducting the study, and reading this section provides very incomplete information.  What exactly is meant by nutritional behaviors?  What does a solution box mean and what does it include?  How useful and sufficient can changing nutritional behaviors be? In the method section, it is first mentioned that the study will be conducted in three stages and the first stage consists of two parts, but steps (two to three) are not properly separated and explained. Following a consistent process in explaining the steps is very helpful in making each step more understandable and might help readability. **For example**: Stage 1: Qualitative Phase Stage 2: Quantitative Phase Stage 3: Solutions Optimization | Lines: 21-45  We thank the reviewer for highlighting these critical points. The Aim section has been revised to:  Define nutritional behaviors: These include dietary choices (e.g., fruit/vegetable intake, consumption of processed foods), meal patterns (e.g., skipping breakfast), and food-related habits (e.g., snacking).  Explain the solution box: It is a toolkit containing evidence-based strategies such as educational pamphlets, interactive activities, and goal-setting templates tailored to the cultural context of Khuzestan province.  Justify the study’s necessity: Poor nutritional behaviors among Iranian adolescent girls are linked to long-term health risks (e.g., obesity, micronutrient deficiencies), making this intervention both timely and relevant. We have restructured the Methods section into three clearly defined stages, each with distinct sub-steps:  Stage 1: Qualitative Phase (focus groups and interviews).  Stage 2: Quantitative Phase (prioritization and ranking of solutions).  Stage 3: Solutions Optimization (matrix development, action plan, and digital visualization).  This reorganization improves readability and ensures a logical flow | **Done** |
| **Background** |  |  |
| line 61 needs a reference. | Thank you. The reference was added | **Done** |
| Line 74-80 needs a reference? | Thank you. The reference was added | **Done** |
| Line 78 to 80, what do you mean by eating behavior? Is it a new factor to be described? | We thank the reviewer for this question. Eating behavior is not a new factor but rather a well-established concept in nutrition and behavioral science. It refers to the combination of dietary choices, attitudes, beliefs, and patterns related to food consumption. In this study, we use the term to capture the psychological and social dimensions of food interactions, which go beyond the repetitive actions described by eating habits or the health-focused aspects of nutritional behaviors. We have revised the text to provide a clearer definition and differentiate eating behavior from related terms. | **Done** |
| Line 83 to 85: These behaviors are associated with a high-risk 84 diet, including inadequate consumption of fruits and vegetables, physical inactivity, insufficient fiber intake, skipping breakfast, and inadequate milk and dairy product consumption,” physical inactivity is not a part of the high-risk diet! | **Thank you. in background the term of** ‘Physical inactivity was omitted | **Done** |
| Line 82 to 89 You can not connect global eating behavior prevalence with childhood obesity prevalence in Iran, Please be more concise and determine what are you exactly trying to report. Line 96 the authors again define eating behaviors with a recent reference, which is almost similar to what they had discussed in line 76. This repetition disrupts consistency in the text and may confuse the reader. It is better to adopt and follow a regular structure in writing the introduction, in such a way that the definitions of eating behaviors, eating habits, eating disorders, and problems are discussed with relevant and correct references, the meaning of eating behaviors is expressed separately in a way that its difference from other meanings is clear, and then regional and global statistics are discussed in a  manner that is exactly in line with the definition. | Thank you for your feedback. We agree with your comments, and the revision has been completed. We have rewritten it for a clearer and more logical flow. | **Done** |
| **Method** |  |  |
| Line133” The philosophical 133 foundation of this research is the pragmatism approach. In this approach, the advantages of both 134 quantitative and qualitative approaches are used to reach the goals of the research” Please make it clear, how can this approach benefit the study.  Line 130:be consistent with the terminology first you say qualitative study, then you talk about qualitative and quantitative approaches! “The mixed method has two 131 phases, and because of the subjectivity of the concept of nutritional behaviors, qualitative study is 132 considered the most suitable method for the first phase of this research. The philosophical 133 foundation of this research is the pragmatism approach. In this approach, the advantages of both 134 quantitative and qualitative approaches are used to reach the goals of the research” | We thank the reviewer for this important query. The pragmatism approach benefits this study by:  **Integrating Qualitative and Quantitative Insights**: Pragmatism allows us to combine in-depth qualitative data (e.g., cultural barriers from interviews) with quantitative prioritization of solutions, ensuring the solution box is both contextually relevant and actionable.  Focusing on Practical Outcomes: Pragmatism prioritizes real-world impact over methodological purity, aligning with our goal of developing practical strategies to improve nutritional behaviors.  Flexibility: It accommodates iterative adjustments to the research design based on emerging findings, enhancing the study’s responsiveness to participant needs.  We appreciate the reviewer’s attention to detail. The text has been revised to consistently use **mixed-methods study** throughout, clarifying that Phase 1 is qualitative and Phase 2 is quantitative. For example: | **Done** |
| Please clarify the difference between lines 147 and 150. | We thank the reviewer for pointing out this lack of clarity. The two bullet points (lines 147–149 and line 150) have been merged into a single, cohesive paragraph that clearly outlines the scope of the literature review. The revised text now distinguishes between:  1. Preventive Nutritional Behaviors: Identifying factors influencing dietary habits and barriers to healthy eating.  2. Best Practices and Interventions: Reviewing evidence-based strategies for improving nutritional behaviors.  This reorganization eliminates redundancy and improves readability. | **Done** |
| Please explain the solution box and how it fits within the study. | We thank the reviewer for this important query. The solution box is a key component of this study, serving as a practical toolkit to improve nutritional behaviors among Iranian female school students. It is developed based on qualitative data collected from students, parents, stakeholders, and decision-makers, analyzed through the lens of Social Cognitive Theory (SCT). The solution box includes evidence-based strategies such as educational materials, interactive tools, and actionable plans tailored to the cultural and socioeconomic context of the target population.  The solution box fits within the study as the primary output, bridging the gap between research findings and real-world application. By addressing both individual (e.g., self-efficacy) and environmental (e.g., peer influence) factors, it provides a comprehensive approach to promoting healthier nutritional behaviors. This ensures that the study’s outcomes are not only theoretically robust but also practical and scalable.  We thank the reviewer for this important query. The solution box is a key component of this study, serving as a practical toolkit to improve nutritional behaviors among Iranian female school students. It is developed based on qualitative data collected from students, parents, stakeholders, and decision-makers, analyzed through the lens of Social Cognitive Theory (SCT). The solution box includes evidence-based strategies such as educational materials, interactive tools, and actionable plans tailored to the cultural and socioeconomic context of the target population.  The solution box fits within the study as the primary output, bridging the gap between research findings and real-world application. By addressing both individual (e.g., self-efficacy) and environmental (e.g., peer influence) factors, it provides a comprehensive approach to promoting healthier nutritional behaviors. This ensures that the study’s outcomes are not only theoretically robust but also practical and scalable. The explanation was added in Visualization of Solutions Box: NUTRIBOX before discussion) lines: 365-389 | **Done** |
| Line 168: Specify data collection and analyzing method” research method using the directed content analysis approach using 169 Social Cognitive Theory constructs” | We thank the reviewer for this suggestion. The revised text now provides a detailed explanation of the data collection and analysis methods | **Done** |
| Line 187: Please provide the ethics approval committee code. | According to PLOSONE submission instruction we are not allowed to mention the ethic code in method section .However is was added in Declaration section under heading **Ethics approval and consent to participate**. | **Explanation** |
| Line 222 needs a reference | The reference for Data Analysis section is supported by Shannon and Hsieh 2005 including | **Done** |
| Line 255. How do you choose the schools?  What is the method of sampling to cover different zones? | Because this comment is similar to reviewer 2 the Method of Data Collection was clarified and revised | **Done** |
| Line 290 needs reference. | Şahin M. A comprehensive analysis of weighting and multicriteria methods in the context of sustainable energy. International Journal of Environmental Science and Technology. 2021 Jun;18(6):1591-616. | **Done** |
| **Discussion** |  |  |
| Considering food security (available and accessible) and its direct impact on dietary habits is crucial. | **Its perfect comment thanks .The** food security was considered in discussion | **Done** |

| **#Reviewer 2** | **Response** | |
| --- | --- | --- |
| The authors have presented a mixed method—qualitative and quantitative—to detect the underlying causes of unhealthy nutritional behaviors in adolescent female students in Khouzestan, Iran. The structured presented follows the rule of logic and shows promising aspects with an important health issue in Iran. However, the following concerns might need to be addressed | Thank you for your encouraging feedback. We are grateful for your thoughtful review and valuable suggestions. Below, we have carefully addressed each point you raised. | |
| **Comments & Suggestions** | **Response** | **Action** |
| **A) Writing/Structure**  1) Line 83: ‘inappropriate’ is not a technical term when talking about eating behaviors. | We thank the reviewer for this observation. The term "inappropriate" has been replaced with **"unhealthy"** to better align with technical terminology in nutrition research. The revised text now reads: "unhealthy eating behaviors, such as excessive consumption of processed foods and sugary beverages." | Done |
| 2) It seems that some words have been missed in Figure 1 under ‘NUTRIBOX.’ | Thank you for your careful review. We acknowledge the typographical error in Figure 1 under ‘NUTRIBOX.’ We have corrected the missing words and revised the figure accordingly. The updated version has been included in the manuscript. | Done |
| 3) Revise the manuscript with regard to its writing. I specifically noticed some misuses of capitalization. | This revision corrects grammar and capitalization errors, and also improves clarity. | Done |
| **B) Methods/Fundamentals**   1) Line 191: Are ‘tape recorders’ still available for use? 2) Are they viable options regarding the existence of digital recorders for safe storage of data? | We thank the reviewer for raising this important point. The term "tape recorders" has been updated to **"digital voice recorders"** to reflect current technology. Digital recorders are more reliable, secure, and widely used in research for data collection. Additionally, we have added a note about data security: "Recordings were stored on password-protected devices and encrypted to ensure confidentiality." This revision ensures compliance with modern research standards and safeguards participant data. | Done |
| 2) Sampling method need more explanation than just to achieve ‘maximum diversity.’ The authors need to specify what schools will be chosen and why. For instance, are schools chosen geographically, based on socio-economic features shared by residing students, etc.? And the method needs to be specified; convenience sampling, cluster sampling, etc. | We thank the reviewers for their valuable feedback. We have revised the Methods section to provide a more detailed explanation of our sampling strategy. As this is a qualitative study, our goal is not generalizability but rather to achieve maximum variation in the sample to capture a wide range of perspectives and experiences. Below, we outline the sampling process:  School Selection: Schools were selected using **purposeful sampling** to ensure representation across different geographic zones (urban, semi-urban, and rural) and socioeconomic status (SES) levels in Andimshek city, Khuzestan province. This approach allows us to capture diverse environmental and cultural contexts that influence nutritional behaviors.  Participant Selection: Within each school, participants (female students, parents, and stakeholders) were selected to reflect diversity in age, education level, SES, and cultural background. We used **stratified purposeful** sampling to ensure inclusion of participants from varying socioeconomic and cultural contexts.  **SES as a Key Criterion** : Socioeconomic status (SES) was explicitly considered during participant selection, as it is a critical factor influencing nutritional behaviors. We categorized participants into low, middle, and high SES groups based on parental education, occupation, and household income.  **Data Saturation**: Recruitment continued until data saturation was achieved, meaning no new themes or insights emerged from the interviews. This ensured depth and richness in the data while maintaining diversity.  These revisions have been incorporated into the Methods section | Done |
| 3) Line 266-267: The fact that the researchers ‘will try to select more diverse participants’ is not the assurance that the authors presume. A detailed plan must be laid out to ensure including students from various backgrounds. I specially recommend that socioeconomic status (SES) of attendees be taken as the main criteria for inclusion of students. In other words, should participants from a specific background be selected—a real possibility with the present scheme—the outputs will lose generalizability. Authors must be aware that eating/nutritional behaviors are widely affected by environmental factors and the present scheme seems to skew towards a major selection bias. |  |  |
| 4) Knowingly, gender and gender identification, as well as issues dealing with sexual activities, commence in adolescence. These subjects, with the potential mediatory impact of distortions in body image or competitive behaviors to create a ‘fitter’ body shape, could modify the individual’s perception of healthy body shape, and thus, their eating behaviors. Are the authors—interviewers—open to discuss these matters with the interviewees? If so, the manuscript needs to include them. | We sincerely thank the reviewer for raising this important point regarding the potential influence of gender identity, sexual activities, and body image on adolescent eating behaviors. We acknowledge that these factors can indeed mediate perceptions of body shape and nutritional behaviors, particularly in the context of adolescence. However, due to socio-cultural and religious sensitivities in the study setting, we are not permitted to directly address or inquire about topics related to sexual activities or gender identity in our interviews with female school students. These constraints are imposed by local ethical guidelines and institutional review boards to ensure the study respects cultural norms and protects participants from discomfort or harm. | Explanation |
