# Supplementary material for: Development of a proposed solutions box for improving nutritional behaviors of female school students: Protocol of a mixed-method study
Source: PLoS One. 2025 Sep 24;20(9):e0329671. doi: 10.1371/journal.pone.0329671 (PMC12459775; doi:10.1371/journal.pone.0329671)
Supplement: Attachment — Submitted filename: REVIEW PLOSONE.docx [file pone.0329671.s003.docx]

**Manuscript Number PONE-D-24-52896R1**

Dear Authors congratulation for your innovative research.

I enjoyed reading it.
