# Supplementary material for: Development of a proposed solutions box for improving nutritional behaviors of female school students: Protocol of a mixed-method study
Source: PLoS One. 2025 Sep 24;20(9):e0329671. doi: 10.1371/journal.pone.0329671 (PMC12459775; doi:10.1371/journal.pone.0329671)
Supplement: Attachment — Submitted filename: Response to reviwer 03.07.2025.doc [file pone.0329671.s005.doc]

**Rebottle letter**

**03.07.2025**

**Dear Academic Editor**

Many thanks for the precise comments on our MS: “**Development of a Proposed Solutions Box for Improving Nutritional Behaviors of Female School Students: Protocol of a Mixed-Method Study.** We greatly appreciate the time and effort you have dedicated to providing us with such valuable feedback.

- We have carefully considered all of the reviewers' comments and have addressed them in tabulated format, which have submitted alongside the revised MS.
- Accordingly, all necessary changes to the MS were **highlighted** in **green**.

Once again, we thank you for your thoughtful review and for helping us to improve our MS.

**Sincerely,**

**Fatemeh Zarei**

Corresponding author

[f.zarei@modares.ac.ir](mailto:f.zarei@modares.ac.ir)

**03.07.2025**

**Response to Reviewers**

**Dear Reviewers,**

We sincerely appreciate your valuable time and insightful comments on our manuscript. Your feedback has significantly improved the quality of our work. We have carefully addressed all your suggestions and revisions, ensuring that each point has been thoroughly considered.

Thank you for your constructive review and support.

**Best regards**

Corresponding Athours

| **#Reviewer 3** | **Response** | |
| --- | --- | --- |
| **Development of a Proposed Solutions Box for Improving Nutritional Behaviors of Female 2 School Students: Protocol of a Mixed-Method Study** **Reviewer #3**: While this article is well-written and informative, and the authors' efforts to provide solutions for improving the nutritional behaviors of female school students are appreciated, a different approach to presenting its unique contributions may be beneficial. | Thank you for your thoughtful feedback. We have carefully addressed your comments and made the necessary revisions, as detailed below. | |
| **Comments & Suggestions** | **Response** | **Action** |
| **Abstract:** 1. The explanation of the aim should be more concise. | We thank the reviewer for this valuable suggestion. We agree that our original aim statement was too detailed and included information better placed elsewhere in the manuscript. To address this, we have revised the aim to be a single, focused sentence. The additional details describing the components of the 'solution box' have been moved to the Methods section, and the broader rationale has been integrated into the Introduction.  **The revised aim now reads:**  'The aim of this study was to develop an evidence-based, context-specific ‘solution box’ to improve the nutritional behaviors of female school students in Khuzestan province  The explanation about the Solution Box mentioned in details in moved **Methods section lines: 378-380** | **Done** |
| 2. Keywords should be verified against MeSH standards. | We thank the reviewer for this valuable suggestion. To addressed this comment all keywords cheaked in Mesh AND the URL of each words was added for consideration  1- Nutritional behavior changed into “Nutrition” that verified in MeSH: <https://www.ncbi.nlm.nih.gov/mesh/?term=Nutrition>  2-Female school students changed into Student : <https://www.ncbi.nlm.nih.gov/mesh/68013334>  3- Feeding Behavior was added : <https://www.ncbi.nlm.nih.gov/mesh/68005247>  4- Qualitative Research  <https://www.ncbi.nlm.nih.gov/mesh/68036301> | **Done** |
| Introduction: 3. The necessity of using a mixed method should be stated more clearly. | "We thank the reviewer for this crucial point. We acknowledge that the rationale for our mixed-methods approach was not sufficiently articulated in the Introduction. To address this, we have now added a dedicated paragraph at the end of the Introduction, just before the statement of the aim, to explicitly justify the use of a sequential exploratory mixed-methods design.  The following paragraph was added in introduction  "Addressing the complexity of nutritional behaviors in this specific cultural context requires a methodology that can both explore nuanced local perspectives and systematically prioritize interventions. A single-method approach would be insufficient; a purely qualitative study could identify potential solutions but would lack a structured mechanism for prioritization, while a purely quantitative study would risk imposing pre-conceived solutions that are not relevant to the lived experiences of students in Khuzestan. Therefore, this study adopts a **sequential exploratory mixed-methods design**. The initial **qualitative phase** is essential to explore and identify culturally-specific barriers and stakeholder-proposed solutions. Subsequently, the **quantitative phase** is necessary to systematically rank and prioritize these qualitatively-derived solutions, building consensus and ensuring feasibility. This integration allows the qualitative findings to directly inform the quantitative instrument, resulting in a final ‘solution box’ that is both empirically robust and contextually grounded." | **Done** |
| Methodology: The methods are adequately described and allow for the replication of the study. | Thanks , we appreciate this feedback |  |
| 4. It seems that a brief explanation of how the results are used in the application should also be given | We thank the reviewer for this excellent suggestion to clarify the practical application of our study's output. We agree that a more detailed description of the final 'solution box' and its use is needed.  To address this, we have expanded upon Stage 3 in the Methods section, renaming it **c) Visualization of Solutions Box: NUTRIBOX**.' This revised section now explicitly describes how the prioritized solutions (the 'results' of the first two stages) will be translated into a user-centered digital toolkit. We have detailed the intended end-users (students, parents, and school staff) and provided concrete examples of the resources that will be developed for each group. This clarifies the pathway from our research findings to a tangible, actionable tool for the community. **So the following paragraph was added in lines: 396-413**  The final stage of this study focuses on translating the research findings into a practical and sustainable application. The "results" from the quantitative prioritization phase (Stage 2) will directly inform the creation of the final 'solution box.' This will not be a static report but a dynamic, user-centered digital toolkit designed for practical implementation by key community members.  The application will be developed as a web-based platform accessible to students, parents, and school health staff. The content will be tailored to each audience:   - **For Female Students:** The platform will feature interactive modules, culturally-appropriate healthy recipes, goal-setting worksheets, and short, engaging videos based on the prioritized solutions. - **For Parents:** It will offer downloadable resources, such as fact sheets on adolescent nutrition, guides for healthy family meals, and strategies to support their children's dietary habits, all grounded in the solutions co-developed with the community. - **For School Staff and Stakeholders:** The toolkit will provide a clear action plan, implementation guides for school-wide initiatives (e.g., healthy canteen policies), and ready-to-use educational materials for classroom integration.   By structuring the application this way, the study ensures that the empirically prioritized solutions are not merely identified but are transformed into an actionable resource that empowers the entire school community to foster healthier nutritional behaviors. | **Done** |
| Discussion: The discussion is well-written and analyzes the implications of the results in light of the existing literature. | Thanks , we appreciate this feedback |  |
| 5. However, it falls short in providing a section on limitations and explaining how the new findings contribute to the existing body of literature. | We sincerely thank the reviewer for pointing out these important omissions. We agree that a discussion of the study's limitations and a clearer statement of its contribution to the literature are essential for a robust protocol. It was added in lines 478 496 | **Done** |
| **Reviewer #4:** Congratulations for your research, I really enjoy reading it. I will recommended to continue with this interesting research line.  Manuscript Number PONE-D-24-52896R1  Dear Authors congratulation for your innovative research.  I enjoyed reading it. | Thanks , we appreciate this feedback |  |
